# Association of Medicare Part D Benzodiazepine Coverage Expansion With Changes in Fall-Related Injuries and Overdoses Among Medicare Advantage Beneficiaries

**DOI:** 10.1001/jamanetworkopen.2020.2051

**Published:** 2020-04-03

**Authors:** Donovan T. Maust, Lewei Allison Lin, Jason E. Goldstick, Rebecca L. Haffajee, Rebecca Brownlee, Amy S. B. Bohnert

**Affiliations:** 1 Injury Prevention Center, University of Michigan, Ann Arbor; 2 Department of Psychiatry, University of Michigan Medical School, Ann Arbor; 3 Center for Clinical Management Research, Veterans Affairs Ann Arbor Healthcare System, Ann Arbor, Michigan; 4 Institute for Healthcare Policy and Innovation, University of Michigan, Ann Arbor; 5 Department of Emergency Medicine, University of Michigan Medical School, Ann Arbor; 6 Department of Health Management and Policy, University of Michigan School of Public Health, Ann Arbor; 7 Economics, Sociology, and Statistics Department, RAND Corporation, Boston, Massachusetts

## Abstract

**Importance:**

Benzodiazepines, which are associated with safety-related harms for older adults, were not covered when the US Medicare Part D prescription drug benefit began. Coverage was extended to benzodiazepines in 2013.

**Objective:**

To examine whether the expansion of benzodiazepine coverage among Medicare Advantage (MA) beneficiaries was associated with increases in fall-related injuries or overdoses among older adults.

**Design, Setting, and Participants:**

This ecological study used interrupted time-series with comparison-series analyses of MA claims data from 4 635 312 age-eligible MA beneficiaries and 940 629 commercially insured individuals (comparison group) stratified by age (65-69, 70-74, 75-79, and ≥80 years) to separately compare trends in fall-related injury and overdose before (January 1, 2010, to December 31, 2012) and after (January 1, 2013, to December 31, 2015) coverage expansion for benzodiazepines. Data analysis was performed from September 1, 2018, to August 31, 2019.

**Exposures:**

Expansion of benzodiazepine coverage in Medicare Part D in 2013.

**Main Outcomes and Measures:**

Monthly rate of fall-related injury and overdose.

**Results:**

In 2012 (the year before the policy change), women constituted 57.5% of the MA group and 47.4% of the comparison group. A total of 25.8% of individuals in the MA group were aged 65 to 69 years, and 29.3% were 80 years or older (mean [SD], 75.1 [6.4] years); 56.7% of individuals in the comparison group were aged 65 to 69 years, and 15.1% were 80 years or older (mean [SD] age, 70.9 [6.5] years). In the MA group, 4 635 312 individuals contributed 156 754 749 person-months from 2010 through 2015; in the comparison group, 940 629 individuals contributed 25 104 534 person-months. After coverage of benzodiazepines began, the rate (ie, slope) of fall-related injury among MA beneficiaries increased from before to after coverage among all age groups. Compared with the comparison group, the increase in rate was statistically significant for those 80 years or older (rate changes for the MA vs comparison groups: 0.12 [95% CI, 0.07 to 0.17] vs −0.01 [95% CI, −0.11 to 0.10]; *P* = .04 for interaction). The overdose trend changed from decreasing to increasing among MA beneficiaries after coverage for all age groups, with a statistically significant increase compared with the comparison group among those aged 65 to 69 years (rate changes for the MA vs comparison groups: 0.23 [95% CI, 0.17 to 0.30] vs 0.02 [95% CI, −0.06 to 0.11]; *P* < .001 for interaction) and among those 80 years or older (rate changes for the MA vs comparison groups: 0.07 [95% CI, 0.00 to 0.14] vs −0.20 [95% CI, −0.35 to −0.05]; *P* = .002 for interaction). Results among MA beneficiaries were consistent when stratified by sex and when limited to those prescribed opioids.

**Conclusions and Relevance:**

Medicare’s expansion of benzodiazepine coverage may have been associated with increases in the rates of overdose among adults ages 65 to 69 years and in the rates of overdose and fall-related injury among those 80 years or older.

## Key Points

**Question:**

Was the 2013 expansion of US Medicare Part D prescription drug coverage to include benzodiazepines associated with increased rates of fall-related injuries or overdoses among older adults?

**Findings:**

This ecological study of more than 4.6 million Medicare Advantage beneficiaries found an increase in the rate of overdose after Part D coverage began to include benzodiazepines among adults aged 65 to 69 years and 80 years or older and an increase in the rate of fall-related injury in adults 80 years or older.

**Meaning:**

Medicare’s expansion of benzodiazepine coverage to older adults may have been associated with an increased rate of overdose among those 69 years or younger and 80 years or older and of injury in those 80 years or older.

## Teaser

This ecological study examines whether the expansion of benzodiazepine coverage among Medicare Advantage beneficiaries was associated with increases in the rates of fall-related injury or overdose among older adults.

## Introduction

Benzodiazepines are associated with a number of safety concerns among older adults, including increased risk of falls and hip fracture.^1,2^ Although overdose receives less attention as a benzodiazepine-related adverse event, benzodiazepines are the second-most common medication class involved in pharmaceutical overdose deaths,^3^ and overdose deaths that involve benzodiazepines increased more than 6-fold from 1996 through 2014.^4^ More than 75% of benzodiazepine-related deaths involve opioids,^3^ and evidence continues to accumulate that use of benzodiazepines is associated with increased risk of opioid-related overdose and mortality.^5,6,7,8^ Given that older adults experienced the largest absolute increases in opioid-related mortality between 2001 and 2016^9^ and also experience the highest rates of coprescribing of opioids and benzodiazepines,^10,11^ benzodiazepine prescribing may be associated with increased opioid-related morbidity and mortality among older adults.

When the Medicare Part D prescription drug coverage program began in 2006, it did not include coverage for benzodiazepines, even though the class is widely prescribed to older adults.^12^ The exclusion was effectively a legacy policy from Medicaid that allowed states to exclude 10 categories of medications from prescription drug coverage, including benzodiazepines.^13^ After Medicare Part D began, there was concern that the lack of benzodiazepine coverage was unduly limiting access to the widely prescribed medication class and causing a financial burden for those who paid out of pocket.^14,15^ In response to these concerns, benzodiazepine coverage was added to Medicare Part D in 2013.^16^ However, lowering the barrier to use of benzodiazepines, which were considered inappropriate for most older adults at the time of the policy change,^17^ may have been associated with safety consequences for the millions of older US individuals enrolled in Medicare Part D plans.

In states that have implemented policies that limit benzodiazepine prescribing, fall-related adverse events among older adults have not decreased.^18,19^ Although restricting benzodiazepine access has not been associated with injury reduction among older adults, less is known about the consequences associated with abruptly expanding benzodiazepine coverage; lowering the threshold for new prescriptions may have a different association with adverse outcomes than restricting coverage. Furthermore, no studies to our knowledge have examined the association of benzodiazepine coverage with overdose among older adults. In this analysis, we examined the association of fall-related injury and overdose with Medicare’s expansion of Part D coverage to include benzodiazepines. Given heightened public attention to opioid prescribing and the potential for harms when opioids and benzodiazepines are coprescribed,^5,8,20^ we also examined outcomes limited to older adults prescribed opioids. We hypothesized that this nationwide expansion of benzodiazepine coverage would be associated with an increase in the rates of fall-related injury and overdose both overall and among those prescribed opioids.

## Methods

### Study Population

For this ecological study, we used administrative health claims from Optum’s deidentified Clinformatics Data Mart Database derived from a large national health insurance company in the US. We created monthly measures of the outcomes using a denominator of all age-eligible (ie, ≥65 years of age) Medicare Advantage (MA) beneficiaries who were enrolled during that month. All older adults in the population had Medicare Part D prescription drug coverage as part of their MA plan. There were no other exclusion criteria. As a comparison group, we used adults 65 years or older in the database with commercial (ie, employer-sponsored) insurance; the prescription plans of these older adults would not have been subject to the benzodiazepine coverage change experienced by MA beneficiaries. The population-level numerators for the 2 analyses were the number of fall-related injury episodes that began in a given month and the number of overdoses. This study was considered not regulated by the Michigan Medicine Institutional Review Board, so informed consent was waived for this analysis of deidentified data. This study followed the Reporting of Studies Conducted Using Observational Routinely Collected Health Data (RECORD) extension of the Strengthening the Reporting of Observational Studies in Epidemiology (STROBE) reporting guideline.^21^

### Outcomes

We conducted separate analyses for the 2 primary outcomes of interest: fall-related injury rates and overdose rates. We also examined changes in benzodiazepine prescribing after the policy change as an intermediary mechanism for the hypothesized changes in our outcomes of interest.

Episodes of fall-related injury were determined using an algorithm developed and validated using MA claims data.^22^ The algorithm captures single episodes of injury (ie, an ED visit, inpatient stay, and rehabilitation visits that followed a single fall event were counted together as a single episode) across the spectrum of severity from nonfracture injury to hip fracture. If a single injury episode crossed calendar months, it was attributed to the month when the episode began.

Overdoses were captured using *International Classification of Diseases, Ninth Revision, Clinical Modification* diagnoses from emergency department or acute inpatient encounters regardless of intent (codes 960-969 and E850-E858 [unintentional], E930-E949 [adverse drug effect], E950.0-E950.5 [self harm], and E980.0-E980.5 [undetermined] based on consensus recommendations from the Injury Surveillance Workgroup^23^).

### Population Characteristics

We obtained information on patient sex and age along with select additional demographic characteristics (eg, US Census division and race/ethnicity) from demographic data available in the Optum database. For descriptive purposes, we also determined the presence of select clinical conditions for which benzodiazepines may commonly be prescribed in clinical practice, including anxiety disorders, depression, dementia, and insomnia (eTable 1 in the Supplement). We stratified these descriptions by insurer type and precoverage and postcoverage period.

### Statistical Analysis

We conducted interrupted time-series analyses to compare the rates of the 2 injury types in the population before and after the Medicare Part D coverage expansion to include benzodiazepines in 2013. For all analyses, the precoverage period was January 1, 2010, to December 31, 2012; the postcoverage period was January 1, 2013, to December 31, 2015. Our models used monthly injury rates as the dependent variable and included a linear time-trend variable, an indicator for the postcoverage period (ie, an indicator for before and after Medicare policy change), and a term for the change in the linear time trend (ie, slope) between the precoverage and postcoverage periods. We did not include a term that tested for an immediate-level shift in outcomes (ie, a change in the model intercept as of the beginning of the postcoverage period in January 2013) because we hypothesized that any change in benzodiazepine prescribing and associated injuries would accumulate over time and not change immediately on January 1, 2013. We constructed analogous models in the comparison group (ie, commercial insurance beneficiaries). To test for the association of the Medicare benzodiazepine coverage policy expansion (ie, a difference in postcoverage rate change between MA beneficiaries and the comparison group), we fit a single model with interactions between group and each term in the interrupted time-series model, specifically reporting the significance of the interaction between groups (MA vs comparison) and the postcoverage rate shift.

Descriptive analyses revealed secular trends in the age distribution of the study population. To guard against confounding on this basis, we stratified analyses by age (65-69, 70-74, 75-79, and ≥80 years). Results of the primary analyses for both outcomes were similar between men and women within each age group; thus, we present primary findings stratified only by age. Models also included monthly fixed effects to account for seasonality of the outcomes. We did not detect any other temporal autocorrelation in the model errors. We used 2-tailed *t* tests to test the statistical significance (α = .05) of the association of benzodiazepine coverage expansion with injury (ie, the rate change between precoverage and postcoverage periods). In addition, given safety concerns about opioid and benzodiazepine coprescribing,^24^ we completed additional analyses that limited the monthly MA population denominator to beneficiaries with 1 day or more of opioid supply in a given month to determine whether results limited to the population prescribed opioids were consistent with those for the overall MA population. Analysis was completed from September 1, 2018, to August 31, 2019.

## Results

In 2012 (the year before the policy change), women constituted 57.5% of the MA group and 47.4% of the comparison group. A total of 25.8% of individuals in the MA group were aged 65 to 69 years, and 29.3% were 80 years or older (mean [SD] age, 75.1 [6.4] years); 56.7% of individuals in the comparison group were aged 65 to 69 years, and 15.1% were 80 years or older (mean [SD] age, 70.9 [6.5] years). There were 4 635 312 individual patients (ie, age-eligible MA beneficiaries), who contributed 156 754 749 person-months from 2010 through 2015. In the comparison group, there were 940 629 individual patients, who contributed 25 104 534 person-months during the same period. Select population characteristics are given in Table 1. There was a marked increase in benzodiazepine claims among MA beneficiaries beginning in 2013; beneficiaries with 1 day or more of benzodiazepine coverage in a given month increased from approximately 0.5% in 2012 to 6% for much of 2013 (eFigure 1 and eFigure 2 in the Supplement).

**Table 1.** Select Sociodemographic and Clinical Characteristics of Medicare Advantage and Commercially Insured Enrollees (Comparison Group) 65 Years or Older During the Year Before and After the 2013 Medicare Part D Policy Change

Characteristic	Study participants, No. (%)			
	2012^a^		2014^a^	
	Medicare Advantage (n = 2 329 980)	Comparison group (n = 442 716)	Medicare Advantage (n = 2 727 897)	Comparison group (n = 395 406)
Age, y				
65-69	601 650 (25.8)	250 848 (56.7)	678 930 (24.9)	248 715 (62.9)
70-74	604 891 (26.0)	80 390 (18.2)	739 907 (27.1)	69 756 (17.6)
75-79	439 890 (18.9)	44 681 (10.1)	514 435 (18.9)	32 484 (8.2)
≥80	683 549 (29.3)	66 797 (15.1)	794 625 (29.1)	44 451 (11.2)
Sex^b^				
Female	1 340 361 (57.5)	209 666 (47.4)	1 577 131 (57.8)	185 400 (46.9)
Male	989 619 (42.5)	232 706 (52.6)	1 150 766 (42.2)	209 588 (53.0)
Race/ethnicity^c^				
Asian	81 133 (3.5)	11 047 (2.50)	98 899 (3.6)	11 451 (2.9)
Black	202 803 (8.7)	34 072 (7.7)	219 710 (8.1)	30 187 (7.6)
Hispanic	222 566 (9.6)	26 677 (6.0)	293 032 (10.7)	28 007 (7.1)
White	1 544 124 (66.3)	351 031 (79.3)	1 765 941 (64.7)	307 435 (77.8)
Unknown	66 483 (2.9)	15 666 (3.5)	80 603 (3.0)	14 259 (3.6)
US Census division				
East North Central	238 417 (10.2)	65 080 (14.7)	374 688 (13.7)	60 583 (15.3)
East South Central	66 568 (2.9)	14 337 (3.2)	69 486 (2.6)	14 467 (3.7)
Middle Atlantic	189 933 (8.2)	29 635 (6.7)	267 295 (9.8)	25 206 (6.4)
Mountain	268 918 (11.5)	46 873 (10.6)	303 652 (11.1)	47 127 (11.9)
New England	131 578 (5.7)	16 185 (3.7)	135 140 (5.0)	14 559 (3.7)
Pacific	471 462 (20.2)	49 625 (11.2)	494 111 (18.1)	45 763 (11.6)
South Atlantic	464 108 (19.9)	103 862 (23.5)	498 123 (18.3)	83 340 (21.1)
West North Central	223 550 (9.6)	61 263 (0.8)	245 803 (9.0)	46 661 (11.8)
West South Central	231 823 (10.0)	52 514 (13.8)	288 748 (10.6)	52 992 (13.4)
Unknown	43 623 (1.9)	3342 (11.9)	50 851 (1.9)	4708 (1.2)
FPL status				
≤400%	1 875 465 (80.5)	404 595 (91.4)	2 185 833 (88.9)	361 352 (91.4)
>400%	84 (0.0)	49 (0.0)	48 (0.0)	50 (0.0)
Unknown or missing	454 431 (19.5)	38 072 (8.6)	542 016 (19.9)	34 004 (8.6)
Select diagnoses				
Any anxiety disorder	56 718 (2.4)	9252 (2.1)	90 219 (3.3)	10 255 (2.6)
Generalized anxiety disorder	51 125 (2.2)	7662 (1.7)	80 149 (2.9)	8334 (2.1)
PTSD	4138 (0.2)	1263 (0.3)	8361 (0.3)	1625 (0.4)
Other anxiety disorders	2714 (0.1)	590 (0.1)	4283 (0.2)	672 (0.2)
Dementia	264 141 (11.3)	25 259 (5.7)	340 597 (12.5)	20 370 (5.2)
Depression	343 778 (14.8)	45 850 (10.4)	485 935 (17.8)	44 818 (11.3)
Seizure disorders	20 469 (0.9)	2802 (0.6)	29 168 (1.1)	2694 (0.7)
Insomnia	184 869 (7.9)	26 565 (6.0)	277 749 (10.2)	28 832 (7.3)

Abbreviations: FPL, federal poverty level; PTSD, posttraumatic stress disorder.

^a^ Includes enrollees with 1 month or more of enrollment during the indicated calendar year.

^b^ Missing for 334 (0.1%) in the 2012 comparison group and 418 (0.1%) in the 2014 comparison group.

^c^ Missing for 212 871 (9.1%) in the Medicare Advantage group in 2012, 269 712 (9.9%) in the Medicare Advantage group in 2014, 4223 (1.0%) in the comparison group in 2012, and 4067 (1.0%) in the comparison group in 2014.

### Fall-Related Injury

**Figure 1.**
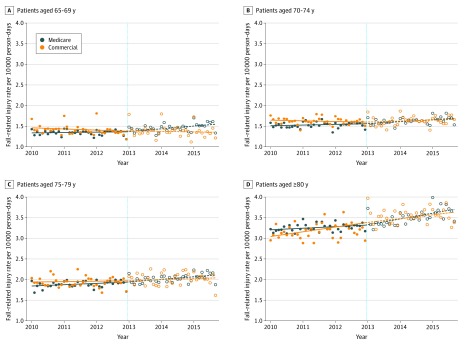
Monthly Rates of Fall-Related Injury Among Medicare Advantage and Commercially Insured Enrollees (Comparison Group) From 2010 to 2015 In models that included fixed effects for month, the interaction term that compared the Medicare Advantage and comparison group rate changes was statistically significant for those 80 years or older (*P* = .04) but not for those aged 65 to 69 years (*P* = .06), 70 to 74 years (*P* = .76), or 75 to 79 years (*P* = .92). Full results are given in Table 2. The vertical dashed line indicates the time of Medicare Part D benzodiazepine coverage expansion.

The baseline rate (ie, the model-predicted rate in January 2010) of fall-related injury was 1.34 per 10 000 person-days (95% CI, 1.31-1.37 per 10 000 person-days) among those aged 65 to 69 years and increased for each age group; the rate was 3.20 per 10 000 person-days (95% CI, 3.15-3.24 per 10 000 person-days) among those 80 years or older (Table 2). Monthly event rates were similar among the comparison group. During the postcoverage period, the rate of fall-related injury increased for all MA age groups (Figure 1). Interaction tests for the rate change between the MA and comparison groups were not significant for the younger age groups (eg, 65-69, 70-74, and 75-79 years). However, for those 80 years or older, the increase in the rate of fall-related injury increased significantly among the MA group compared with the comparison group during the postcoverage period (rate changes for the MA vs comparison groups: 0.12 [95% CI, 0.07 to 0.17] vs −0.01 [95% CI, −0.11 to 0.10]; *P* = .04 for interaction). The MA analyses stratified by sex yielded similar results for men and women by age group (eTable 2 in the Supplement).

**Table 2.** Interrupted Time-Series Analysis of Monthly Fall-Related Injury Among MA and Commercially Insured Enrollees (Comparison Group) From 2010 to 2015

Group by age^a^	Before coverage (2010–2012)			After coverage (2013–2015)			Before vs after coverage		
	Fall-related injury per 10 000 person-days (95% CI)^b^	Rate (95% CI)^c^	*P* value^c^	Fall-related injury per 10 000 person-days (95% CI)^b^	Rate (95% CI)^c^	*P* value^c^	Rate change (95% CI)	*P* value	*P* value for MA vs commercial rate change
65-69 y									
MA (n = 3 547 378)	1.34 (1.31 to 1.37)	0.00 (−0.01 to 0.02)	.76	1.38 (1.33 to 1.42)	0.06 (0.03 to 0.09)	.001	0.05 (0.02 to 0.09)	.002	.06
Comparison (n = 815 567)	1.44 (1.41 to 1.48)	−0.02 (−0.04 to 0.00)	.05	1.40 (1.34 to 1.45)	−0.02 (−0.05 to 0.02)	.33	0.01 (−0.03 to 0.04)	.76	
70-74 y									
MA (n = 1 668 561)	1.50 (1.47 to 1.54)	0.02 (0.00 to 0.04)	.10	1.56 (1.52 to 1.59)	0.05 (0.02 to 0.07)	<.001	0.03 (0.00 to 0.06)	.08	.76
Comparison (n = 211 269)	1.62 (1.58 to 1.67)	0.00 (−0.02 to 0.03)	.91	1.57 (1.50 to 1.64)	0.04 (0.00 to 0.08)	.09	0.04 (−0.01 to 0.09)	.13	
75-79 y									
MA (n = 1 211 833)	1.84 (1.80 to 1.89)	0.03 (0.00 to 0.05)	.07	1.95 (1.90 to 1.99)	0.06 (0.03 to 0.09)	.001	0.03 (−0.01 to 0.07)	.13	.92
Comparison (n = 107 596)	1.98 (1.91 to 2.06)	−0.03 (−0.07 to 0.02)	.24	2.02 (1.90 to 2.13)	0.00 (−0.07 to 0.07)	.99	0.03 (−0.06 to 0.11)	.51	
≥80 y									
MA (n = 1 439 617)	3.20 (3.15 to 3.24)	0.04 (0.01 to 0.06)	.006	3.30 (3.23 to 3.37)	0.16 (0.11 to 0.20)	<.001	0.12 (0.07 to 0.17)	<.001	.04
Comparison (n = 118 640)	3.12 (2.99 to 3.25)	0.04 (−0.04 to 0.11)	.36	3.51 (3.39 to 3.63)	0.03 (−0.05 to 0.11)	.47	−0.01 (−0.11 to 0.10)	.91	

Abbreviation: MA, Medicare Advantage.

^a^ Sample size reflects total number from 2010 to 2015.

^b^ Data from the first month of the period.

^c^ All rate, rate changes, and *P* values reported were obtained from regression models that included fixed effects for month. The rate presents the change per month in the injury rate.

Among MA beneficiaries prescribed opioids, the fall rate was markedly higher than in the overall population; however, the precoverage and postcoverage changes were consistent (eTable 3 in the Supplement). Similar to the main analysis, the rate of fall-related injury changed after coverage; the precoverage rate was either negative or flat and shifted to an increasing trend in all age groups.

### Overdose

**Figure 2.**
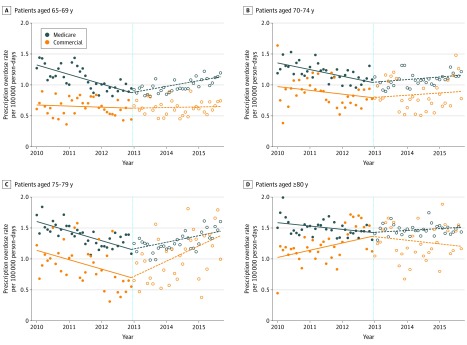
Monthly Rates of Overdose Among Medicare Advantage and Commercially Insured Enrollees From 2010 to 2015 In models that included fixed effects for month, the interaction term that compared the Medicare Advantage and comparison group rate changes was statistically significant for those aged 65 to 69 years (*P* < .001) and those 80 years or older (*P* = .002) and not significant for those age 70 to 74 years (*P* = .33) or 75 to 79 years old (*P* = .49). Full results are given in Table 3. The vertical dashed line indicates the time of Medicare Part D benzodiazepine coverage expansion.

The baseline rate (ie, the model-predicted rate in January 2010) of overdose was 1.31 per 100 000 person-days (95% CI, 1.22-1.40 per 100 000 person-days) among those aged 65 to 69 years. The rate was highest among those aged 75 to 79 years (1.61 per 10 000 person-days; 95% CI, 1.52-1.70 per 100 000 person-days) (Table 3). The overdose rate was lower among the comparison group for all ages. During the postcoverage period (2013-2015), the rate of overdose among MA beneficiaries increased for all age groups (Figure 2). Compared with the comparison group, the increases in postcoverage overdose rates were statistically significant among those 65 to 69 years old and among those 80 years or older. For those aged 65 to 69 years, the postcoverage rate increased by 0.23 encounters per month (95% CI, 0.17-0.30) vs 0.02 (95% CI, −0.06 to 0.11; *P* < .001 for interaction) for the comparison group. For those 80 years or older, the postcoverage rate increased by 0.07 encounters per month (95% CI, 0.00-0.14 encounters per month) vs −0.20 (95% CI, −0.35 to −0.05; *P* = .002 for interaction) for the comparison group. The MA analyses stratified by sex yielded similar results for men and women by age group (eTable 4 in the Supplement).

**Table 3.** Interrupted Time-Series Analysis of Monthly Overdose Among MA and Commercially Insured Enrollees (Comparison Group) From 2010 to 2015

Group by age^a^	Before coverage (2010–2012)			After coverage (2013–2015)			Before vs after coverage change		
	Overdose per 100 000 person-days (95% CI)^b^	Rate (95% CI)^c^	*P* value^c^	Overdose per 100 000 person-days (95% CI)^b^	Rate (95% CI)^c^	*P* value^c^	Rate change (95% CI)	*P* value	*P* value for MA vs commercial rate change
65-69 y									
MA (n = 3 547 378)	1.31 (1.22 to 1.40)	−0.14 (−0.19 to −0.09)	<.001	0.86 (0.81 to 0.91)	0.10 (0.06 to 0.13)	<.001	0.23 (0.17 to 0.30)	<.001	<.001
Comparison (n = 815 567)	0.67 (0.57 to 0.78)	−0.02 (−0.08 to 0.04)	.57	0.63 (0.54 to 0.72)	0.01 (−0.05 to 0.07)	.83	0.02 (−0.06 to 0.11)	.57	
70-74 y									
MA (n = 1 668 561)	1.35 (1.27 to 1.43)	−0.10 (−0.14 to −0.05)	<.001	1.02 (0.95 to 1.09)	0.05 (0.01 to 0.10)	.05	0.15 (0.08 to 0.21)	<.001	.33
Comparison (n = 211 269)	1.00 (0.84 to 1.16)	−0.08 (−0.17 to 0.01)	.11	0.84 (0.67 to 1.02)	−0.01 (−0.12 to 0.10)	.88	0.07 (−0.07 to 0.21)	.35	
75-79 y									
MA (n = 1 211 833)	1.61 (1.52 to 1.70)	−0.14 (−0.20 to −0.09)	<.001	1.10 (1.01 to 1.19)	0.13 (0.07 to 0.19)	<.001	0.27 (0.19 to 0.35)	<.001	.46
Comparison (n = 107 596)	1.18 (0.95 to 1.41)	−0.18 (−0.31 to −0.04)	.01	0.80 (0.54 to 1.05)	0.18 (0.01 to 0.34)	.049	0.36 (0.14 to 0.57)	.002	
≥80 y									
MA (n = 1 439 617)	1.60 (1.51 to 1.69)	−0.06 (−0.12 to −0.01)	.03	1.47 (1.41 to 1.53)	0.00 (−0.03 to 0.04)	.87	0.07 (0.00 to 0.14)	.06	.002
Comparison (n = 118 640)	0.91 (0.76 to 1.05)	0.21 (0.13 to 0.29)	<.001	1.24 (1.03 to 1.44)	0.01 (−0.12 to 0.14)	.89	−0.20 (−0.35 to −0.05)	.01	

Abbreviation: MA, Medicare Advantage.

^a^ Sample size reflects total number from 2010 to 2015.

^b^ Data from the first month of the period.

^c^ All rate, rate changes, and *P* values reported were obtained from regression models that included fixed effects for month. The rate presents the change per month in the injury rate.

As with the fall rate, the monthly rate of overdose was markedly higher among beneficiaries prescribed opioids than in the overall population (eTable 5 in the Supplement). Similar to the main analysis, the monthly event rate reversed direction from decreasing before coverage to increasing after coverage for those aged 65 to 69 years and 75 to 79 years, with a nonsignificant change among those aged 70 to 74 years.

## Discussion

In this analysis of 4.6 million MA beneficiaries, we found that, at the population level, expansion of Medicare Part D coverage to include benzodiazepines was associated with increases in benzodiazepine prescription claims for all adults 65 years or older. Our findings suggest that there may have been associated increases in the rate of injury among those aged 65 to 69 years and those 80 years or older. The oldest group experienced increasing rates of both fall-related injury and overdose, whereas among those aged 65 to 69 years, the trend of overdose increased. Findings were not different when the population was limited to older adults who were prescribed opioids.

Although the increase in benzodiazepine claims among MA beneficiaries was increased by 5.5% or more than 10-fold, there were some benzodiazepine claims during the precoverage period (ie, 2010-2012). This finding likely reflects that some MA plans could provide more generous prescription coverage than required by Medicare that included benzodiazepines.^25^ The increase in claims for benzodiazepines likely overestimates the true increase in exposure among older adults because benzodiazepines are available in generic, inexpensive forms; some patients were likely self-paying for benzodiazepine prescriptions before the 2013 coverage change. In an analysis of MA beneficiaries in plans that included prescription drug coverage (including benzodiazepines) before Medicare Part D existed but then excluded benzodiazepines when Medicare Part D began, nearly 75% of MA beneficiaries continued use of benzodiazepines after exclusion by paying out of pocket.^15^ In addition, a recent analysis^4^ of benzodiazepine prescribing trends from 1996 to 2014 using the Medical Expenditure Panel Survey did not demonstrate an increase in prescription benzodiazepine use among respondents 65 years or older in 2013.^4^

We hypothesized that the expansion of coverage would be associated with both types of injury. However, the minimal effect sizes observed align with prior studies^18,19^ of policies that restricted benzodiazepine access that did not demonstrate a decrease in injury. One such analysis^19^ that predated Medicare Part D examined hip fracture rates, comparing New York, which instituted a triplicate prescription policy that increased the administrative burden of benzodiazepine prescribing, with New Jersey, which had no such policy. Although there was an immediate 60% reduction in benzodiazepine prescribing in New York, the rate of hip fracture did not decrease compared with that in New Jersey. When Medicare Part D went into effect, some state Medicaid programs provided supplemental coverage of benzodiazepines, whereas other states did not. In an analysis^18^ of dually eligible nursing home residents, benzodiazepine prescribing varied with states’ supplemental coverage, but the risk of hip fracture was not lower in states that limited benzodiazepine coverage.^18^

Given these prior analyses, what may account for the limited associations with increased injury in the present analysis? The expansion of Medicare Part D may have had a limited association with overall prevalence of benzodiazepine use among older adults but still would have been associated with a reduced threshold for new use among benzodiazepines-naive patients, particularly those with limited resources. Because those who regularly use benzodiazepines develop physiologic tolerance with repeated exposure,^26^ new users may be particularly susceptible to experiencing an injury when first exposed. A study by Hernandez et al^27^ examining the risk of opioid-related overdose among concurrent opioid and benzodiazepine users suggests that the risk of overdose is highest in the first 90 days of concurrent use and then decreases with each successive time increment.^27^ In addition, some practitioners and patients may have interpreted Medicare’s benzodiazepine coverage expansion as an indication that safety risks had been overstated, further lowering the prescribing threshold and leading to prescriptions that practitioners previously avoided because of safety concerns.

We did not anticipate that the change in the overdose rate associated with benzodiazepine coverage would be greater in relative terms than the change in fall-related injury. Safety warnings cautioning against benzodiazepine prescribing in older adults typically focus on the risk of falls.^28^ Their association with overdose other than when prescribed with opioids receives less attention, and unlike for fall-related injury, we are unaware of prior analyses examining the association of benzodiazepine coverage policies with overdose. This lack of attention may be partially because overdose is less common among adults 65 years or older than among younger adults^29^ and is therefore not perceived as a problem. However, a recent analysis^9^ found that the largest relative increases in opioid-related overdose from 2001 to 2016 were among adults aged 55 to 64 years followed by those 65 years or older.

Why might the associations with increased injury have been limited to those aged 65 to 69 years and those 80 years or older in the study population? It is possible that these age groups were vulnerable to the effects of policy change for different reasons. Adverse effects related to benzodiazepines are generally attributed to the role of benzodiazepine as a central nervous system depressant, and older adults are particularly sensitive to these effects.^30,31^ An 80-year-old individual may be more sensitive to these effects than a 65-year-old individual. The 65- to 69-year age group includes members of the baby boom cohort, who started turning 65 years of age in 2011. Compared with prior cohorts of older adults (ie, the other age groups in this analysis), baby boomers have higher rates of alcohol use, nonmedical prescription opioid use, and marijuana use,^32,33,34,35^ each of which might be associated with increased susceptibility to benzodiazepine-related injury from a new prescription. By occurring at the height of the opioid overdose crisis, the benzodiazepine coverage expansion potentially exacerbated the risks associated with prescription opioids.

We did not expect the overdose rate to have decreased during the precoverage period; across the US overall, overdose-related deaths among those 65 years or older increased slightly during that time.^29^ Although favorable selection into MA plans has decreased,^36,37^ evidence remains that patients with the most complex needs may exit MA,^38,39^ and MA patients at the end of life are healthier, more educated, and have fewer functional limitations.^40^ The rate also decreased among the comparison group except among those 80 years or older. The population of adults 65 years or older who continue to maintain employment with employer-sponsored insurance coverage may be healthier and are likely not representative of the general older adult population.

### Limitations

This study has limitations. First, the study was limited to MA beneficiaries and not the overall population with Medicare Part D coverage; thus, our findings may not generalize to the entire population of older adults. Second, claims data have limited ability to detect and appropriately classify both of the primary outcomes, which thus may have been limited to events serious enough to require medical attention and to those appropriately coded by practitioners. Third, we cannot account for the true prevalence of benzodiazepine use in the population because patients were likely paying for prescriptions out of pocket or receiving coverage through other sources (eg, Veterans Affairs or Medicaid). Fourth, we did not adjust for patient-level characteristics that may have been changing during this time and potentially contributed to increased falls or overdoses, although our findings remained when restricted to those prescribed opioids.

## Conclusions

Compared with commercial coverage among similarly aged older adults, Medicare’s expansion of benzodiazepine coverage to older adults may have been associated with an increased rate of overdose among those 69 years or younger and 80 years or older and of injury in those 80 years or older. To our knowledge, this was the first analysis to consider the association of benzodiazepine coverage policy with the risk of overdose.

## Notes

Supplement. **eTable 1. ***ICD-9-CM* Codes Used to Derive Select Clinical Characteristics **eTable 2.** Interrupted Time Series Analysis of Monthly Fall-Related Injury Among Medicare Advantage Beneficiaries Stratified by Age and Sex, 2010–2015 **eTable 3.** Fall-Related Injury Among Medicare Advantage Beneficiaries Prescribed Opioids Stratified by Age, 2010–2015 **eTable 4.** Overdose Among Medicare Advantage Beneficiaries Stratified by Age and Sex, 2010–2015 **eTable 5.** Overdose Among Medicare Advantage Beneficiaries Prescribed Opioids Stratified by Age, 2010–2015 **eFigure 1.** Monthly Proportions of Medicare Advantage and Commercially-Insured Enrollees (Comparison Group) With ≥1 Day of Benzodiazepine Available, 2010–2015 **eFigure 2.** Monthly Proportions of Medicare Advantage and Commercially-Insured Enrollees (Comparison Group) With ≥1 Day of Benzodiazepine Available Stratified by Age, 2010–2015

## References

[1] Tinetti ME, Speechley M, Ginter SF Risk factors for falls among elderly persons living in the community. N Engl J Med. 1988;319(26):-. doi:10.1056/NEJM198812293192604 3205267

[2] Ray WA, Griffin MR, Downey W Benzodiazepines of long and short elimination half-life and the risk of hip fracture. JAMA. 1989;262(23):3303-3307. doi:10.1001/jama.1989.03430230088031 2573741

[3] Jones CM, Mack KA, Paulozzi LJ Pharmaceutical overdose deaths, United States, 2010. JAMA. 2013;309(7):657-659. doi:10.1001/jama.2013.272 23423407

[4] Bachhuber MA, Hennessy S, Cunningham CO, Starrels JL Increasing benzodiazepine prescriptions and overdose mortality in the United States, 1996-2013. Am J Public Health. 2016;106(4):686-688. doi:10.2105/AJPH.2016.303061 26890165 PMC4816010

[5] Park TW, Saitz R, Ganoczy D, Ilgen MA, Bohnert ASB Benzodiazepine prescribing patterns and deaths from drug overdose among US veterans receiving opioid analgesics: case-cohort study. BMJ. 2015;350:h2698. doi:10.1136/bmj.h2698 26063215 PMC4462713

[6] Cho J, Spence MM, Niu F, Hui RL, Gray P, Steinberg S Risk of overdose with exposure to prescription opioids, benzodiazepines, and non-benzodiazepine sedative-hypnotics in adults: a retrospective cohort study. J Gen Intern Med. Published online January 9, 2020. doi:10.1007/s11606-019-05545-y 31919729 PMC7080944

[7] Burke LG, Zhou X, Boyle KL, Trends in opioid use disorder and overdose among opioid-naive individuals receiving an opioid prescription in Massachusetts from 2011 to 2014. Addiction. 2020;115(3):493-504. doi:10.1111/add.14867 31691390

[8] Sun EC, Dixit A, Humphreys K, Darnall BD, Baker LC, Mackey S Association between concurrent use of prescription opioids and benzodiazepines and overdose: retrospective analysis. BMJ. 2017;356:j760. doi:10.1136/bmj.j760 28292769 PMC5421443

[9] Gomes T, Tadrous M, Mamdani MM, Paterson JM, Juurlink DN The burden of opioid-related mortality in the United States. JAMA Netw Open. 2018;1(2):e180217. doi:10.1001/jamanetworkopen.2018.0217 30646062 PMC6324425

[10] Zhang VS, Olfson M, King M Opioid and benzodiazepine coprescribing in the United States before and after US Food and Drug Administration boxed warning. JAMA Psychiatry. 2019;76(11):1208-1210. doi:10.1001/jamapsychiatry.2019.2563 31532463 PMC6751777

[11] Guy GP Jr, Zhang K, Halpin J, Sargent W An examination of concurrent opioid and benzodiazepine prescribing in 9 states, 2015. Am J Prev Med. 2019;57(5):629-636. doi:10.1016/j.amepre.2019.06.007 31564606 PMC6917208

[12] Qato DM, Wilder J, Schumm LP, Gillet V, Alexander GC Changes in prescription and over-the-counter medication and dietary supplement use among older adults in the United States, 2005 vs 2011. JAMA Intern Med. 2016;176(4):473-482. doi:10.1001/jamainternmed.2015.8581 26998708 PMC5024734

[13] Bambauer KZ, Sabin JE, Soumerai SB The exclusion of benzodiazepine coverage in Medicare: simple steps for avoiding a public health crisis. Psychiatr Serv. 2005;56(9):1143-1146. doi:10.1176/appi.ps.56.9.1143 16148332

[14] Chen H, Nwangwu A, Aparasu R, Essien E, Sun S, Lee K The impact of Medicare Part D on psychotropic utilization and financial burden for community-based seniors. Psychiatr Serv. 2008;59(10):1191-1197. doi:10.1176/ps.2008.59.10.1191 18832506

[15] Ong MK, Xu H, Zhang L, Azocar F, Ettner SL Effect of Medicare Part D benzodiazepine exclusion on psychotropic use in benzodiazepine users. J Am Geriatr Soc. 2012;60(7):1292-1297. doi:10.1111/j.1532-5415.2012.04031.x 22725849 PMC4387568

[16] Centers for Medicare & Medicaid Services Transition to Part D coverage of benzodiazepines and barbiturates beginning in 2013 Accessed May 6, 2016. https://www.cms.gov/Medicare/Prescription-Drug-Coverage/PrescriptionDrugCovContra/Downloads/BenzoandBarbituratesin2013.pdf

[17] American Geriatrics Society 2012 Beers Criteria Update Expert Panel American Geriatrics Society updated Beers Criteria for potentially inappropriate medication use in older adults. J Am Geriatr Soc. 2012;60(4):616-631. doi:10.1111/j.1532-5415.2012.03923.x 22376048 PMC3571677

[18] Briesacher BA, Soumerai SB, Field TS, Fouayzi H, Gurwitz JH Medicare Part D’s exclusion of benzodiazepines and fracture risk in nursing homes. Arch Intern Med. 2010;170(8):693-698. doi:10.1001/archinternmed.2010.57 20421554 PMC2907144

[19] Wagner AK, Ross-Degnan D, Gurwitz JH, Effect of New York State regulatory action on benzodiazepine prescribing and hip fracture rates. Ann Intern Med. 2007;146(2):96-103. doi:10.7326/0003-4819-146-2-200701160-00004 17227933

[20] Hanlon JT, Boudreau RM, Roumani YF, Number and dosage of central nervous system medications on recurrent falls in community elders: the Health, Aging and Body Composition study. J Gerontol A Biol Sci Med Sci. 2009;64(4):492-498. doi:10.1093/gerona/gln043 19196642 PMC2657172

[21] Benchimol EI, Smeeth L, Guttmann A, ; RECORD Working Committee The Reporting of studies Conducted using Observational Routinely-collected health Data (RECORD) statement. PLoS Med. 2015;12(10):e1001885. doi:10.1371/journal.pmed.1001885 26440803 PMC4595218

[22] Kim S-B, Zingmond DS, Keeler EB, Development of an algorithm to identify fall-related injuries and costs in Medicare data. Inj Epidemiol. 2016;3(1):1-13. doi:10.1186/s40621-015-0066-z 27747538 PMC4701758

[23] Injury Surveillance Workgroup Consensus Recommendations for National and State Poisoning Surveillance. Safe States Alliance; 2012.

[24] US Food and Drug Administration FDA Drug Safety Communication: FDA warns about serious risks and death when combining opioid pain or cough medicines with benzodiazepines; requires its strongest warning. August 31, 2016 Accessed May 10, 2019. https://www.fda.gov/media/99761/download

[25] Centers for Medicare & Medicaid Services Important information about Medicare coverage of drugs under Part B and the new Medicare prescription drug coverage (Part D), and vaccines administered in a physician’s office—the ninth in the MLN Matters series on the new prescription drug plans. Accessed July 23, 2019. https://www.cms.gov/outreach-and-education/medicare-learning-network-mln/mlnmattersarticles/downloads/SE0570.pdf

[26] O’Brien CP Benzodiazepine use, abuse, and dependence. J Clin Psychiatry. 2005;66(suppl 2):28-33. 15762817

[27] Hernandez I, He M, Brooks MM, Zhang Y Exposure-response association between concurrent opioid and benzodiazepine use and risk of opioid-related overdose in Medicare Part D beneficiaries. JAMA Netw Open. 2018;1(2):e180919-e180919. doi:10.1001/jamanetworkopen.2018.0919 30646080 PMC6324417

[28] By the 2019 American Geriatrics Society Beers Criteria® Update Expert Panel American Geriatrics Society 2019 updated AGS Beers Criteria® for potentially inappropriate medication use in older adults. J Am Geriatr Soc. 2019;67(4):674-694. doi:10.1111/jgs.15767 30693946

[29] Hedegaard H, Warner M, Minino AM Drug overdose deaths in the United States, 1999-2015. NCHS Data Brief. 2017;(273):1-8. 28256996

[30] Turnheim K When drug therapy gets old: pharmacokinetics and pharmacodynamics in the elderly. Exp Gerontol. 2003;38(8):843-853. doi:10.1016/S0531-5565(03)00133-5 12915206

[31] Tannenbaum C, Paquette A, Hilmer S, Holroyd-Leduc J, Carnahan R A systematic review of amnestic and non-amnestic mild cognitive impairment induced by anticholinergic, antihistamine, GABAergic and opioid drugs. Drugs Aging. 2012;29(8):639-658. 22812538 10.1007/BF03262280

[32] Soldo BJ, Mitchell OS, Tfaily R, McCabe J Cross-cohort differences in health on the verge of retirement. National Bureau of Economic Research; December 2006. Working paper 12762.

[33] Blazer DG, Wu L-T The epidemiology of substance use and disorders among middle aged and elderly community adults: national survey on drug use and health. Am J Geriatr Psychiatry. 2009;17(3):237-245. doi:10.1097/JGP.0b013e318190b8ef 19454850 PMC2721326

[34] Blazer DG, Wu L-T Nonprescription use of pain relievers by middle-aged and elderly community-living adults: National Survey on Drug Use and Health. J Am Geriatr Soc. 2009;57(7):1252-1257. doi:10.1111/j.1532-5415.2009.02306.x 19486199 PMC2752277

[35] Han BH, Moore AA, Sherman S, Keyes KM, Palamar JJ Demographic trends of binge alcohol use and alcohol use disorders among older adults in the United States, 2005-2014. Drug Alcohol Depend. 2017;170:198-207. doi:10.1016/j.drugalcdep.2016.11.003 27979428 PMC5241162

[36] McWilliams JM, Hsu J, Newhouse JP New risk-adjustment system was associated with reduced favorable selection in Medicare Advantage. Health Aff (Millwood). 2012;31(12):2630-2640. doi:10.1377/hlthaff.2011.1344 23213147 PMC3538078

[37] Newhouse JP, Price M, Huang J, McWilliams JM, Hsu J Steps to reduce favorable risk selection in Medicare Advantage largely succeeded, boding well for health insurance exchanges. Health Aff (Millwood). 2012;31(12):2618-2628. doi:10.1377/hlthaff.2012.0345 23213145 PMC3535470

[38] Meyers DJ, Belanger E, Joyce N, McHugh J, Rahman M, Mor V Analysis of drivers of disenrollment and plan switching among Medicare Advantage beneficiaries. JAMA Intern Med. 2019;179(4):524-532. doi:10.1001/jamainternmed.2018.7639 30801625 PMC6450306

[39] Rahman M, Keohane L, Trivedi AN, Mor V High-cost patients had substantial rates of leaving Medicare Advantage and joining traditional Medicare. Health Aff (Millwood). 2015;34(10):1675-1681. doi:10.1377/hlthaff.2015.0272 26438743 PMC4676406

[40] Byhoff E, Harris JA, Ayanian JZ Characteristics of decedents in Medicare Advantage and traditional Medicare. JAMA Intern Med. 2016;176(7):1020-1023. doi:10.1001/jamainternmed.2016.2266 27273237

