# Supplementary material for: Association of Medicare Part D Benzodiazepine Coverage Expansion With Changes in Fall-Related Injuries and Overdoses Among Medicare Advantage Beneficiaries
Source: JAMA Netw Open. 2020 Apr 3;3(4):e202051. doi: 10.1001/jamanetworkopen.2020.2051 (PMC7125434; doi:10.1001/jamanetworkopen.2020.2051)
Supplement: Supplement. — eTable 1. ICD-9-CM Codes Used to Derive Select Clinical Characteristics eTable 2. Interrupted Time Series Analysis of Monthly Fall-Related Injury Among Medicare Advantage Beneficiaries Stratified by Age and Sex, 2010–2015 eTable 3. Fall-Related Injury Among Medicare Advantage Beneficiaries Prescribed Opioids Stratified by Age, 2010–2015 eTable 4. Overdose Among Medicare Advantage Beneficiaries Stratified by Age and Sex, 2010–2015 eTable 5. Overdose Among Medicare Advantage Beneficiaries Prescribed Opioids Stratified by Age, 2010–2015 eFigure 1. Monthly Proportions of Medicare Advantage and Commercially-Insured Enrollees (Comparison Group) With ≥1 Day of Benzodiazepine Available, 2010–2015 eFigure 2. Monthly Proportions of Medicare Advantage and Commercially-Insured Enrollees (Comparison Group) With ≥1 Day of Benzodiazepine Available Stratified by Age, 2010–2015 [file jamanetwopen-3-e202051-s001.pdf]

Supplementary Online Content

Maust DT, Lin LA, Goldstick JE, Haffajee RL, Brownlee R, Bohnert ASB. Association of Medicare Part D benzodiazepine coverage expansion with changes in fall-related injuries and overdoses among Medicare Advantage beneficiaries. *JAMA Netw Open*. 2020;3(4):e202051. doi:10.1001/jamanetworkopen.2020.2051

eTable 1. *ICD-9-CM* Codes Used to Derive Select Clinical Characteristics
eTable 2. Interrupted Time Series Analysis of Monthly Fall-Related Injury Among Medicare Advantage Beneficiaries Stratified by Age and Sex, 2010–2015

eTable 3. Fall-Related Injury Among Medicare Advantage Beneficiaries Prescribed Opioids Stratified by Age, 2010–2015

eTable 4. Overdose Among Medicare Advantage Beneficiaries Stratified by Age and Sex, 2010–2015

eTable 5. Overdose Among Medicare Advantage Beneficiaries Prescribed Opioids Stratified by Age, 2010–2015

eFigure 1. Monthly Proportions of Medicare Advantage and Commercially-Insured Enrollees (Comparison Group) With ≥1 Day of Benzodiazepine Available, 2010–2015

eFigure 2. Monthly Proportions of Medicare Advantage and Commercially-Insured Enrollees (Comparison Group) With ≥1 Day of Benzodiazepine Available Stratified by Age, 2010–2015

This supplementary material has been provided by the authors to give readers additional information about their work.

© 2020 Maust DT et al. *JAMA Network Open.*

eTable 1. ***ICD-9-CM*** **codes used to derive select clinical characteristics**

**Clinical characteristic**	***ICD-9-CM*** **codes**
Anxiety disorders	
Generalized anxiety disorder	300.02
PTSD	309.81
Other anxiety disorders	300.0, 300.3
Dementia	290.0, 290.1-290.13, 290.20, 290.21, 290.3, 290.40-290.43, 294.10, 294.11, 294.20, 294.21, 294.8, 331.0,331.11, 331.19, 331.2-331.7, 331.81-331.83, 331.89, 331.9, 797
Depression	296.20-296.26, 296.30-296.36, 300.4, 311
Seizure disorders	345.00 345.01, 345.10, 345.11, 345.2, 345.3, 345.40, 345.41, 345.50, 345.51, 345.70, 345.71, 345.80, 345.81, 345.9, 345.91
Insomnia	307.40-307.49, 327.00-327.02, 327.09, 327.42, 780.51, 780.52

© 2020 Maust DT et al. *JAMA Network Open.*

eTable 2. Interrupted time series analysis of monthly fall-related injury among Medicare Advantage beneficiaries stratified by age and sex, 2010–2015

	Pre-coverage (2010–2012)			Post-coverage (2013–2015)			Pre- to Post-Coverage Change in Slope[a]	
	**Fall-related injury per 10,000 person-days (95% CI)[b]**	**Slope (95% CI)[c]**	**P value[c]**	**Fall-related injury per 10,000 person-days (95% CI)[b]**	**Slope (95% CI)**	**P value**	**Slope change (95% CI)**	**P value**
65-69	1.34 (1.31, 1.37)	0.00 (-0.01, 0.02)	0.76	1.38 (1.33, 1.42)	0.06 (0.03, 0.09)	0.001	0.05 (0.02, 0.09)	0.002
Female	1.55 (1.52, 1.59)	0.01 (-0.01, 0.03)	0.56	1.62 (1.57, 1.67)	0.05 (0.02, 0.09)	0.007	0.05 (0.01, 0.08)	0.02
Male	1.07 (1.04, 1.10)	-0.01 (-0.02, 0.01)	0.56	1.07 (1.02, 1.11)	0.06 (0.03, 0.08)	<0.001	0.06 (0.03, 0.09)	<0.001
70-74	1.50 (1.47, 1.54)	0.02 (0.00, 0.04)	0.10	1.56 (1.52, 1.59)	0.05 (0.02, 0.07)	<0.001	0.03 (0.00, 0.06)	0.08
Female	1.77 (1.73, 1.82)	0.02 (0.00, 0.05)	0.10	1.85 (1.80, 1.89)	0.04 (0.01, 0.07)	0.01	0.02 (-0.02, 0.06)	0.29
Male	1.17 (1.14, 1.21)	0.01 (-0.01, 0.04)	0.18	1.21 (1.18, 1.24)	0.05 (0.03, 0.07)	<0.001	0.03 (0.00, 0.06)	0.04
75-79	1.84 (1.80, 1.89)	0.03 (0.00, 0.05)	0.07	1.95 (1.90, 1.99)	0.06 (0.03, 0.09)	0.001	0.03 (-0.01, 0.07)	0.13
Female	2.19 (2.14, 2.25)	0.03 (0.00, 0.06)	0.10	2.31 (2.24, 2.38)	0.06 (0.01, 0.10)	0.02	0.03 (-0.03, 0.08)	0.33
Male	1.39 (1.34, 1.45)	0.03 (-0.01, 0.06)	0.12	1.49 (1.45, 1.53)	0.06 (0.03, 0.08)	<0.001	0.03 (-0.01, 0.07)	0.16
80+	3.20 (3.15, 3.24)	0.04 (0.01, 0.06)	0.006	3.30 (3.23, 3.37)	0.16 (0.11, 0.20)	<0.001	0.12 (0.07, 0.17)	<0.001
Female	3.65 (3.61, 3.70)	0.06 (0.03, 0.08)	<0.001	3.78 (3.70, 3.85)	0.17 (0.12, 0.22)	<0.001	0.11 (0.06, 0.17)	<0.001
Male	2.41 (2.36, 2.47)	0.03 (-0.01, 0.06)	0.14	2.53 (2.45, 2.60)	0.13 (0.08, 0.18)	<0.001	0.11 (0.05, 0.16)	<0.001

CI: confidence interval

[a] Policy change is the Medicare Part D expanded coverage of benzodiazepines starting in January 2013.

[b] During first month of the period.

[c] All slope, slope changes, and P values reported were obtained from regression models that included fixed effects for month.

© 2020 Maust DT et al. *JAMA Network Open.*

eTable 3. Interrupted time series analysis of monthly fall-related injury among Medicare Advantage beneficiaries prescribed opioids stratified by age, 2010–2015

	Pre-coverage (2010–2012)			Post-coverage (2013–2015)			Pre- to Post-Coverage Change in Slope[a]	
	**Fall-related injury per 10,000 person-days (95% CI)[b]**	**Slope (95% CI)[c]**	**P value[c]**	**Fall-related injury per 10,000 person-days (95% CI)[b]**	**Slope (95% CI)**	**P value**	**Slope change (95% CI)**	**P value**
65-69	5.70 (5.49, 5.90)	-0.09 (-0.21, 0.03)	0.16	5.71 (5.53, 5.90)	0.16 (0.04, 0.28)	0.01	0.25 (0.08, 0.42)	0.005
70-74	6.15 (5.98, 6.33)	-0.05 (-0.15, 0.05)	0.34	6.02 (5.86, 6.19)	0.20 (0.09, 0.30)	0.002	0.25 (0.10, 0.40)	0.002
75-79	7.07 (6.80, 7.33)	-0.03 (-0.18, 0.12)	0.68	6.94 (6.69, 7.18)	0.15 (-0.01, 0.31)	0.09	0.18 (-0.04, 0.40)	0.12
80+	9.84 (9.65, 10.02)	-0.12 (-0.23, -0.02)	0.03	9.48 (9.30, 9.67)	0.26 (0.14, 0.38)	<0.001	0.38 (0.22, 0.54)	<0.001

CI: confidence interval

[a] Policy change is the Medicare Part D expanded coverage of benzodiazepines starting in January 2013.

[b] During first month of the period.

[c] All slope, slope changes, and P values reported were obtained from regression models that included fixed effects for month.

© 2020 Maust DT et al. *JAMA Network Open.*

eTable 4. Interrupted time series analysis of monthly overdose among Medicare Advantage beneficiaries stratified by age and sex, 2010–2015

	Pre-coverage (2010–2012)			Post-coverage (2013–2015)			Pre- to Post-Coverage Change in Slope[a]	
	**Overdose per 100,000 person-days (95% CI)[b]**	**Slope (95% CI)[c]**	**P value[c]**	**Overdose per 100,000 person-days (95% CI)[b]**	**Slope (95% CI)**	**P value**	**Slope change (95% CI)**	**P value**
65-69	1.31 (1.22, 1.40)	-0.14 (-0.19, -0.09)	<0.001	0.86 (0.81, 0.91)	0.10 (0.06, 0.13)	<0.001	0.23 (0.17, 0.30)	<0.001
Female	1.32 (1.19, 1.45)	-0.08 (-0.16, -0.01)	0.04	0.98 (0.91, 1.05)	0.06 (0.02, 0.11)	0.01	0.15 (0.06, 0.24)	0.003
Male	1.31 (1.20, 1.42)	-0.21 (-0.27, -0.15)	<0.001	0.71 (0.64, 0.79)	0.13 (0.08, 0.18)	<0.001	0.34 (0.26, 0.42)	<0.001
70-74	1.35 (1.27, 1.43)	-0.10 (-0.14, -0.05)	<0.001	1.02 (0.95, 1.09)	0.05 (0.01, 0.10)	0.05	0.15 (0.08, 0.21)	<0.001
Female	1.47 (1.37, 1.57)	-0.12 (-0.17, -0.06)	<0.001	1.18 (1.09, 1.28)	0.00 (-0.06, 0.06)	0.89	0.12 (0.04, 0.20)	0.007
Male	1.21 (1.10, 1.32)	-0.07 (-0.13, -0.01)	0.03	0.81 (0.72, 0.90)	0.11 (0.05, 0.17)	0.002	0.18 (0.10, 0.27)	<0.001
75-79	1.61 (1.52, 1.70)	-0.14 (-0.20, -0.09)	<0.001	1.10 (1.01, 1.19)	0.13 (0.07, 0.19)	<0.001	0.27 (0.19, 0.35)	<0.001
Female	1.57 (1.45, 1.70)	-0.10 (-0.18, -0.03)	0.01	1.19 (1.06, 1.33)	0.11 (0.02, 0.20)	0.02	0.21 (0.10, 0.33)	<0.001
Male	1.65 (1.44, 1.86)	-0.19 (-0.32, -0.07)	0.004	0.98 (0.87, 1.09)	0.15 (0.07, 0.22)	<0.001	0.34 (0.19, 0.49)	<0.001
80+	1.60 (1.50, 1.69)	-0.06 (-0.12, -0.01)	0.03	1.47 (1.41, 1.53)	0.00 (-0.03, 0.04)	0.87	0.07 (0.00, 0.14)	0.06
Female	1.60 (1.50, 1.70)	-0.05 (-0.11, 0.00)	0.09	1.48 (1.38, 1.57)	0.02 (-0.04, 0.09)	0.51	0.08 (-0.01, 0.16)	0.10
Male	1.60 (1.46, 1.73)	-0.08 (-0.16, -0.01)	0.04	1.46 (1.34, 1.58)	-0.03 (-0.10, 0.05)	0.05	0.05 (-0.05, 0.16)	0.03

CI: confidence interval

[a] Policy change is the Medicare Part D expanded coverage of benzodiazepines starting in January 2013.

[b] During first month of the period.

[c] All slope, slope changes, and P values reported were obtained from regression models that included fixed effects for month.

© 2020 Maust DT et al. *JAMA Network Open.*

eTable 5. Interrupted time series analysis of monthly overdose among Medicare Advantage beneficiaries prescribed opioids stratified by age, 2010–2015

	Pre-coverage (2010–2012)			Post-coverage (2013–2015)			Pre- to Post-Coverage Change in Slope[a]	
	**Overdose per 100,000 person-days (95% CI)[b]**	**Slope (95% CI)[c]**	**P value[c]**	**Overdose per 100,000 person-days (95% CI)[b]**	**Slope (95% CI)**	**P value**	**Slope change (95% CI)**	**P value**
65-69	4.59 (4.19, 4.99)	-0.27 (-0.50, -0.04)	0.03	3.62 (3.21, 4.04)	0.31 (0.05, 0.58)	0.03	0.59 (0.24, 0.94)	0.002
70-74	3.84 (3.43, 4.25)	0.05 (-0.18, 0.29)	0.66	3.69 (3.25, 4.12)	0.19 (-0.10, 0.47)	0.21	0.13 (-0.23, 0.50)	0.48
75-79	4.86 (4.39, 5.33)	-0.42 (-0.68, -0.15)	0.01	3.48 3.03, 3.92)	0.34 (0.06, 0.63)	0.03	0.76 (0.36, 1.15)	<0.001
80+	3.44 (3.09, 3.79)	0.22 (0.02, 0.42)	0.04	4.01 (3.58, 4.43)	-0.13 (-0.40, 0.15)	0.37	-0.35 (-0.68, -0.01)	0.05

CI: confidence interval

[a] Policy change is the Medicare Part D expanded coverage of benzodiazepines starting in January 2013.

[b] During first month of the period.

[c] All slope, slope changes, and P values reported were obtained from regression models that included fixed effects for month.

© 2020 Maust DT et al. *JAMA Network Open.*

eFigure 1. Monthly proportions of Medicare Advantage and commercially-insured enrollees (comparison group) with ≥1 day of benzodiazepine available, 2010–2015

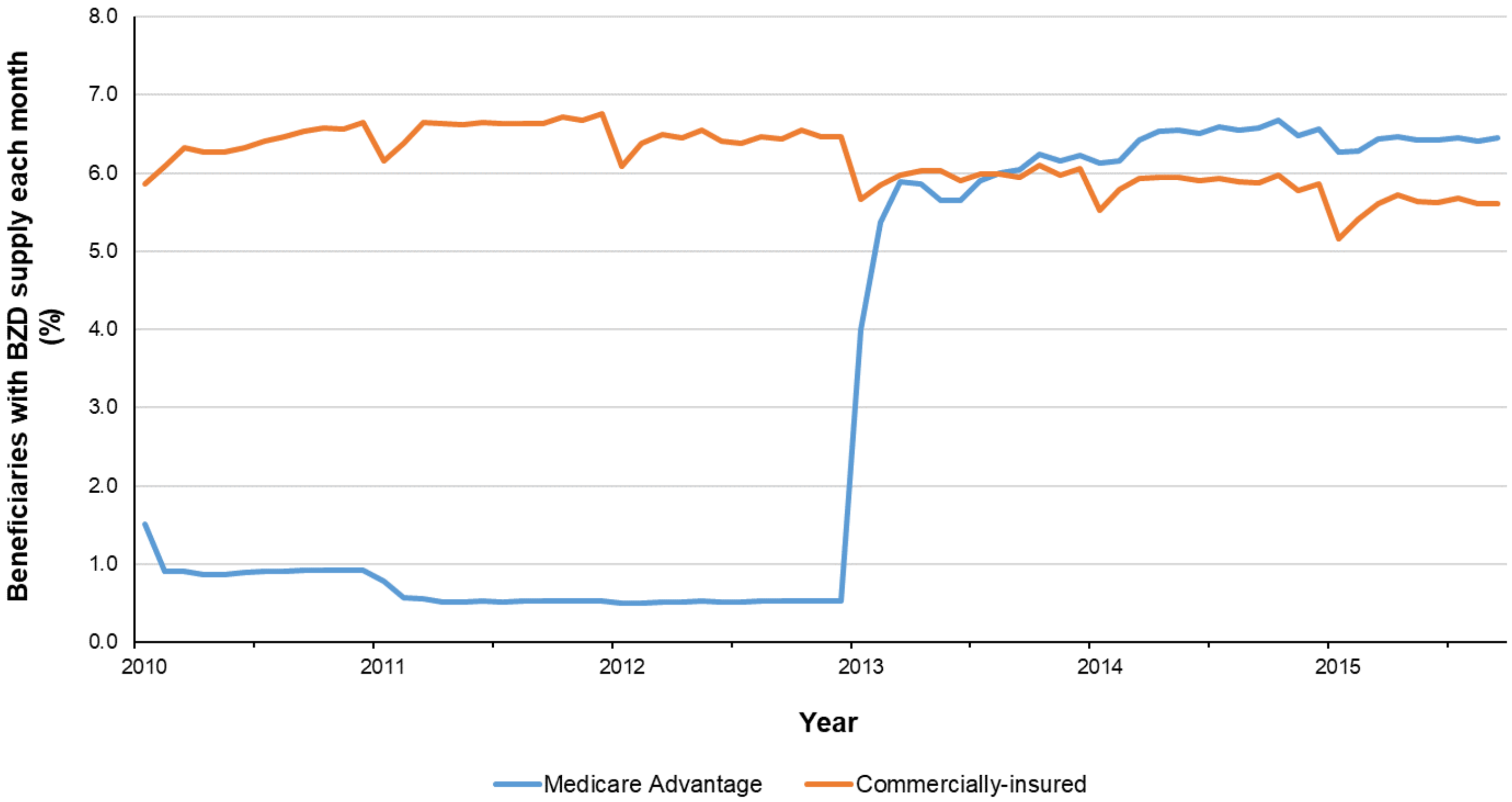

The date of the prescription claim and days'-supply field were used to determine whether beneficiaires possessed benzodiazepines in a given month.

© 2020 Maust DT et al. *JAMA Network Open.*

eFigure 2. Monthly proportions of Medicare Advantage and commercially-insured enrollees (comparison group) with ≥1 day of benzodiazepine available stratified by age, 2010–2015

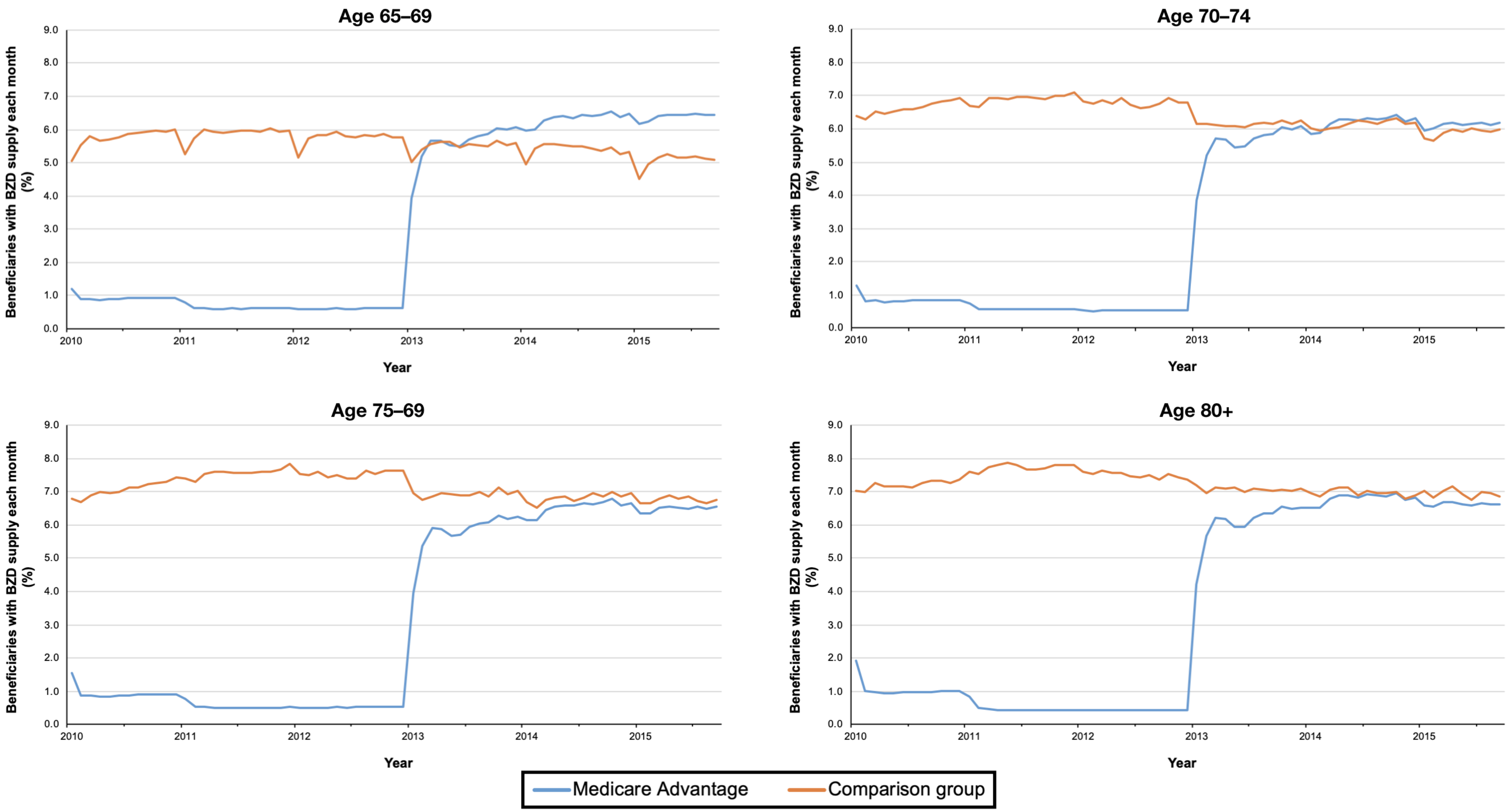

© 2020 Maust DT et al. *JAMA Network Open.*

© 2020 Maust DT et al. *JAMA Network Open.*
